# Acceptance of AI-Powered Chatbots Among Physiotherapy Students: International Cross-Sectional Study

**DOI:** 10.2196/76574

**Published:** 2025-08-19

**Authors:** Salwa B El-Sobkey, Kerolous Ishak Kelini, Mahmoud ElKholy, Tayseer Abdeldayem, Mariam Abdallah, Dina Al-Amir Mohamed, Aya Fawzy, Yomna F Ahmed, Ayman El Khatib, Hind Khalid, Balkhis Banu Shaik, Ana Anjos, Mutasim D Alharbi, Karim Fathy, Khaled Takey

**Affiliations:** 1 Department of Physiotherapy, Fatima College of Health Sciences, Khalifa Bin Zayed Street, Al Maqam, PO Box 24162, Al Ain, Abu Dhabi, United Arab Emirates, 971 509287399; 2 Faculty of Physical Therapy, Beni-Suef University, Beni-Suef, Egypt; 3 Department of Physical Therapy, Faculty of Applied Medical Sciences, Al-Zaytoonah University of Jordan, Amman, Jordan; 4 Faculty of Physical Therapy, Modern University for Technology and Information, Cairo, Egypt; 5 Physical Therapy Department, Faculty of Health Sciences, Beirut Arab University, Beirut, Lebanon; 6 Faculty of Physical Therapy, October 6 University, October 6, Egypt; 7 Department of Physical Therapy, Faculty of Medical Rehabilitation Sciences, King Abdulaziz University, Jeddah, Saudi Arabia; 8 Faculty of Physical Therapy, Misr University for Sciences and Technology, October 6, Egypt

**Keywords:** artificial intelligence, AI-powered chatbots, health care students, physiotherapy education, TAM, technology acceptance model, technology acceptance

## Abstract

**Background:**

Artificial intelligence–powered chatbots (AI-PCs) are increasingly integrated into educational settings, including health care disciplines. Despite their potential to enhance learning, limited research has investigated physiotherapy (PT) students’ acceptance of this technology.

**Objective:**

This study aims to assess undergraduate PT students’ acceptance of AI-PCs and to identify personal, academic, and technological factors influencing their acceptance.

**Methods:**

Over a 4-month period, a cross-sectional survey was conducted across 7 PT programs in 5 countries. Eligible participants were national undergraduate PT students. The technology acceptance model (TAM)–based questionnaire was used for capturing perceived usefulness, perceived ease of use, attitude, behavioral intention, and actual behavioral use of AI-PCs. The influence of personal, academic, and technological factors was examined. Descriptive and inferential statistics were conducted.

**Results:**

The mean total TAM score was 3.59 (SD 0.82), indicating moderate acceptance. Of the 1066 participants, 375 (35.2%) showed high acceptance, 650 (60.9%) moderate, and 41 (3.9%) low. Prior experience with artificial intelligence (AI) tools emerged as the strongest predictor of acceptance (β=.43; *P*<.001), followed by university affiliation (ANOVA *P*<.001). Cumulative grade point average percentage was positively correlated with TAM score (*r*=0.135; *P*<.001) but was not a significant predictor in regression (*P*=.23). Age (*P*=.54), sex (*P*=.56), academic level (*P*=.26), and current use of AI-PCs (*P*=.10) were not significant predictors.

**Conclusions:**

PT students demonstrated moderate acceptance of AI-PCs. Prior technological experience was the strongest predictor, underscoring the importance of early exposure to AI tools. Educational institutions should consider integrating AI technologies to enhance students’ familiarity and foster positive attitudes toward their use.

## Introduction

### Background

Artificial intelligence (AI) enables computer systems to imitate human intelligence by processing external data and learning to perform specific tasks [1-4]. Large language models, a type of AI, are trained for human-like communication, with artificial intelligence–powered chatbots (AI-PCs) being one of their key applications [5-7]. AI has gained significant attention in health care and health professional education due to its potential to make learning more accessible, affordable, and effective [8,9]. Universities are increasingly adopting AI technologies, with AI-PC use expected to grow as technology advances [6,10-12].

In the 1970s, chatbots were known as pedagogical agents [13] and are now referred to as conversational agents, tutors, or simply bots [14]. Literature identifies AI-PCs as valuable educational tools that enhance learning experiences and outcomes [6,15,16], though they also face challenges such as accuracy, bias, and user attitudes [14,17]. The acceptance of AI-PCs in education remains underexplored, with a need for further research to clarify their adoption in universities [16,18]. To examine students’ acceptance of AI-PCs, it is important to use a well-established technology acceptance model (TAM) [19].

TAM, originally developed by Davis in 1989 and updated in 1993 [20,21], is the most widely used framework for studying technology acceptance in education [22,23]. It is considered a reliable source for understanding the students’ acceptance of learning technologies due to its educational focus and robust structure [24,25]. According to TAM, technology acceptance follows a 3-stage process, in which external factors trigger cognitive responses, perceived usefulness (PU) and perceived ease of use (PEU), that subsequently shape affective responses, namely attitude toward using technology and behavioral intention (BI), ultimately resulting in actual behavioral use (ABU) [20,21].

The PU refers to the individual’s beliefs that a technology will enhance task performance, while PEU reflects how effortless it is to use [26,27]. While BI and attitude represent the potential consequences of the behavior [28], ABU is the outcome [23]. Investigating external factors such as students’ age and prior knowledge could provide valuable insights [6].

AI is among the top 10 priorities for physiotherapy (PT) service improvement [29], and while its role in treatment is documented, research on its application in PT education remains scarce [5]. Further studies are required to explore the role of AI-PCs in educational settings and external factors influencing their adoption [6]. Moreover, understanding how students’ characteristics interact with AI-PCs is crucial for educators and policy makers.

### Objective

Building on this need, this study aims to assess undergraduate PT students’ acceptance of AI-PCs and to identify personal, academic, and technological factors influencing their acceptance.

## Methods

### Ethical Considerations

Ethics approval was obtained from the respective institutional research boards in each participating institution: Fatima College of Health Sciences (FCHS) Institutional Research Board (approval: FECE-1-24-25ELSOBKEY), Beni-Suef University (BSU) Ethical Committee (approval: 12492024), Al-Zaytoonah University of Jordan (ZUJ) Institutional Research Board (approval: 10/10/2024‐2025), Modern University for Technology and Information (MTI) Research Ethical Committee (approval: REC/2111/MTI.PT/2410292), Beirut Arab University (BAU) Institutional Research Board (approval: 2024-H-0206-HS-M-0643), October 6 University (O6U) Research Ethical Committee (approval: O6U.PT.REC/024/002009), and King Abdulaziz University (KAU) Research Ethics Committee (approval: 320‐24). The students’ confidentiality measures were explained in a participant information sheet. The questionnaire was anonymous, and the researchers are the only authorized body to have access to the collected data. Additionally, it was emphasized that participation was entirely voluntary, without academic consequences for nonparticipation. Participants signed a consent form, which was provided at the end of the participant’s information sheet. For digital surveying, the following statement was included in the first section of the Google Forms: “Responding to this questionnaire will be considered as agreement to participate in the study.”

### Study Design

This study used an international cross-sectional survey design. The study was conducted across 7 PT programs in 5 countries (Table 1). Data were collected from September 29, 2024, to January 26, 2025. Undergraduate PT students in the participating universities were the target population and were recruited through bulletin boards, college emails, WhatsApp messages, and verbal invitations. A participant information sheet was distributed for paper-based surveying and was the first section on the digital questionnaire. It explained the study’s purpose, procedure, and the average time to respond (7, SD 1.5 minutes). Convenience sampling was used. Eligible participants were national students enrolled in a bachelor-level PT program who voluntarily provided informed consent. Exclusion criteria included digital or hybrid learning enrollment, repeating multiple courses, being at the internship level (as not all programs included this level), or not completing the questionnaire. All eligible students were invited to participate.

**Table 1.** Countries, Universities, physiotherapy programs, and number of participating students (N=1066).

Country and university		College or department	Participating students, n (%)
1. Egypt			
	Beni-Suef University	Faculty of Physical Therapy	200 (18.8)
	October 6 University	Faculty of Physical Therapy	200 (18.8)
	Modern University for Technology and Information	Faculty of Physical Therapy	200 (18.8)
2. United Arab Emirates			
	Institute of Applied Technology	Physiotherapy Department, Fatima College of Health Sciences	107 (10.0)
3. Saudi Arabia			
	King Abdulaziz University	Department of Physical Therapy, Faculty of Medical Rehabilitation Sciences	67 (6.3)
4. Lebanon			
	Beirut Arab University	Physical Therapy Department, Faculty of Health Sciences	200 (18.8)
5. Jordan			
	Al‐Zaytoonah University of Jordan	Department of Physical Therapy- Faculty of Applied Medical Sciences	92 (8.6)

### Variables

Variables were aligned with TAM constructs and relevant external factors. Outcome variables included the TAM 5 constructs: PU, defined as students’ perception of AI-PCs’ benefits to their studies; PEU, referring to how easy students find AI-PCs to use; attitude, representing students’ overall feelings toward AI-PCs; BI, assessed as the intention to use AI-PCs among nonusers and the intention to continue use among current users; and ABU, measuring AI-PC use. A total TAM score was calculated as the average of these 5 constructs. External factors included personal: age and sex; academic: university affiliation, academic level, cumulative grade point average percentage (CGPA%), and current use of AI-PCs; and technological: prior experience with technologies or AI tools other than AI-PCs.

### Data Collection Process

Students first answered an initial screening question about their prior experience with AI-PCs to determine which version of the questionnaire to complete. Those with experience received questionnaire A, and those without experience received modified questionnaire B. For the digital version, researchers provided clear instructions and links, highlighting the importance of selecting the appropriate questionnaire according to prior AI-PCs’ use. The digital questionnaire back button character was enabled to allow respondents to review and change their answers. To avoid multiple entries from the same student, the form setting of a limit to 1 response was activated.

### Data Sources or Measurement

Two structured questionnaires were used. Questionnaire A (for students who are currently using or who had previously used AI-PCs) included assessed external factors (7 items) and the basic TAM section (24 items, covering 5 constructs, scored by a 5-point Likert scale) [20,21,27]. Instructions were adapted from Lewis [27]. Mean scores were calculated for each construct and overall acceptance, with scores ranging from 1 to 5. Students were categorized into high (≥4), moderate (2‐3.9), and low (<2) acceptance. Questionnaire B (for students with no prior AI-PC experience) followed the same structure but excluded the ABU construct. Both questionnaires were anonymous and were available in digital (Google Forms) and paper-based formats. The digital questionnaire was an open survey for students who received the link from the corresponding investigators. In both questionnaire versions, students were instructed that the questionnaire is divided into 2 sections, and they need to respond to each question by choosing the most accurate answer that reflects their personal experience or opinion. The 5-point Likert scale was also explained, and if they have any questions or concerns, they can freely contact the research team at their institution.

### Pilot Study

The questionnaire was piloted with at least 5 students per institution. Feedback led to 1 minor revision from King Abdulaziz University. Completion time ranged from 5 to 15 minutes (average 7, SD 1.5 minutes). The final version was used in both formats. Additionally, the digital questionnaire was tested for usability and technical functionality.

### Efforts to Address Potential Sources of Bias

Student enrollment varied widely across institutions, ranging from approximately 100 to 1000s of PT students, posing a risk of statistical bias. To reduce overrepresentation, all eligible students from smaller institutions were included, while 200 students were sampled from larger ones. Additionally, to ensure curricular consistency, only bachelor’s students were included, excluding those in Doctor of Physiotherapy programs due to structural and competency differences. Internship-level students were also excluded, as not all institutions offered this academic level. These measures aimed to enhance sample homogeneity and minimize variability that could affect the study’s outcomes.

Further, only national students were included to minimize cultural and linguistic variability across diverse settings, as such cross-cultural differences may influence students’ acceptance of AI-PCs. Similarly, students who were repeating more than 1 course during the data collection semester were excluded, as repeated exposure to content may lead to an inflated perception of the chatbot’s usefulness and a biased response in TAM-related items. These measures were taken to enhance sample homogeneity and reduce variability that could affect the study’s outcomes.

### Handling of Data

Digital responses were exported to a Microsoft Excel sheet, and paper-based data were entered manually. All entries were reviewed for accuracy before statistical analysis.

### Statistical Methods

Descriptive statistics included means and SDs for continuous variables and frequency distributions for categorical variables. Inferential tests included the chi-square test and Pearson or Spearman correlations to assess associations and correlations between total TAM score, acceptance categories, and external factors. An independent 2-tailed *t* test compared TAM scores between users and nonusers. ANOVA with Tukey post hoc tests examined differences in TAM scores, construct scores, CGPA%, and prior experience across universities. The chi-square test also assessed differences in categorical variables like acceptance categories and academic level across universities. Finally, linear regression identified significant predictors of AI-PCs and measured effect sizes. The threshold for statistical significance was set at *P*<.05.

### Handling Missing Data

All items of the digital questionnaire were mandatory to be answered to enable the questionnaire submission. Instead, a few number of students, fewer than 10 students, answered by placing a dot for age and cumulative grade point average (CGPA) items. Missing data for these 2 items were replaced with mean values calculated separately for each institution.

### Reporting

This study was reported by the STROBE (Strengthening the Reporting of Observational Studies in Epidemiology) checklist for cross-sectional studies in addition to the CHERRIES (Checklist for Reporting Results of Internet E-Surveys) [30].

## Results

### Participants

A total of 1066 PT students participated in the study (Table 1). Four institutions (BSU, O6U, MTI, and BAU) each contributed 200 students, meeting the target sample size. The remaining 3 institutions (FCHS, ZUJ, and KAU) had fewer participants but achieved a 100% response rate among eligible students. These institutions follow a 4-year study plan, unlike the 5-year structure on the other sites. At FCHS and ZUJ, many students were nonnationals and excluded based on eligibility criteria. At KAU, 67 students participated, as PT specialization begins in the second year; this number represented the entire eligible cohort.

### Demographic and Academic Characteristics

Table 2 presents the demographic and academic characteristics of participating PT students. The mean age was 20.1 (SD 1.5) years, and 64.1% (n=683) were female. Students were enrolled across all academic years, with the highest proportion in the second year (n=367, 34.4%) and the lowest in the fifth year (n=103, 9.7%). The fifth year applies only to Egyptian universities (BSU, O6U, and MTI), while others follow a 4-year program. Due to differences in CGPA scales (4.0 vs 5.0) across universities, CGPA% was used for standardization, resulting in a mean of 79.7% (SD 14%). CGPA% was not applicable for first-year students in their first semester, as their CGPA was not yet available.

**Table 2.** Demographic, academic, and technological characteristics of the participating physiotherapy students (N=1066).

Characteristic		Values
Age (years)		
	Mean (SD)	20.1 (1.5)
	Range	17.0-28.0
Cumulative grade point average percentage (n=924)		
	Mean (SD)	79.7 (14.0)
	Range	26.8-100.0
Sex, n (%)		
	Female	683 (64.1)
	Male	383 (35.9)
Academic level, n (%)		
	First year	176 (16.5)
	Second year	367 (34.4)
	Third year	237 (22.2)
	Fourth year	183 (17.2)
	Fifth year	103 (9.7)
Use of artificial intelligence–powered chatbots, n (%)		
	Yes	586 (55.0)
	No	480 (45.0)

### PT Students’ Acceptance of AI-PCs

PT students’ acceptance of AI-PCs, as measured by the TAM, had a mean total score of 3.59 of 5 (SD 0.81), reflecting overall moderate acceptance across all universities. The mean scores for the 5 TAM constructs were: PU=3.69 (SD 0.92), PEU=3.68 (SD 0.88), attitude=3.58 (SD 0.91), BI=3.57 (SD 0.91), and ABU=3.40 (SD 0.91). Based on TAM categories, 35.2% (n=375) of students demonstrated high acceptance, 60.9% (n=649) moderate, and 3.9% (n=42) low.

### External Factors Influencing PT students’ Acceptance of AI-PCs

Personal, academic, and technological factors were assessed as potential predictors of acceptance of AI-PCs. Among personal factors, age (Table 3) showed no significant correlation with the TAM mean score (*P*=.54), and sex showed no significant association with TAM acceptance categories (*P*=.56).

**Table 3.** Correlation between physiotherapy students’ total mean score of technology acceptance of artificial intelligence–powered chatbots (AI-PCs) and external factors (N=1066).

External factors	*r*	*P* value
TAM^a^ total score of AI-PCs*students’ age	−0.019	.54
TAM total score of AI-PCs*students’ CGPA%^b^	0.135	<.001
TAM total score of AI-PCs*students’ previous experience of using other artificial intelligence–powered learning tools or applications beyond chatbots	0.445	<.001

a TAM: technology acceptance model.

b CGPA%: cumulative grade point average percentage.

**Figure 1.**
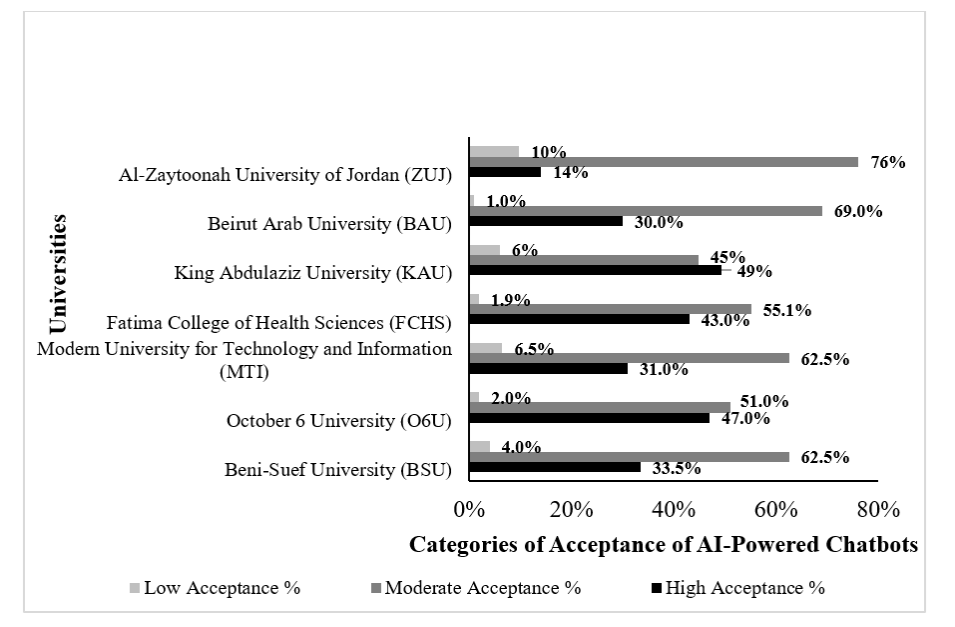
Distribution of physiotherapy students’ acceptance categories of AI-powered chatbots across universities (N=1066; *P*<.001). AI: artificial intelligence.

**Figure 2.**
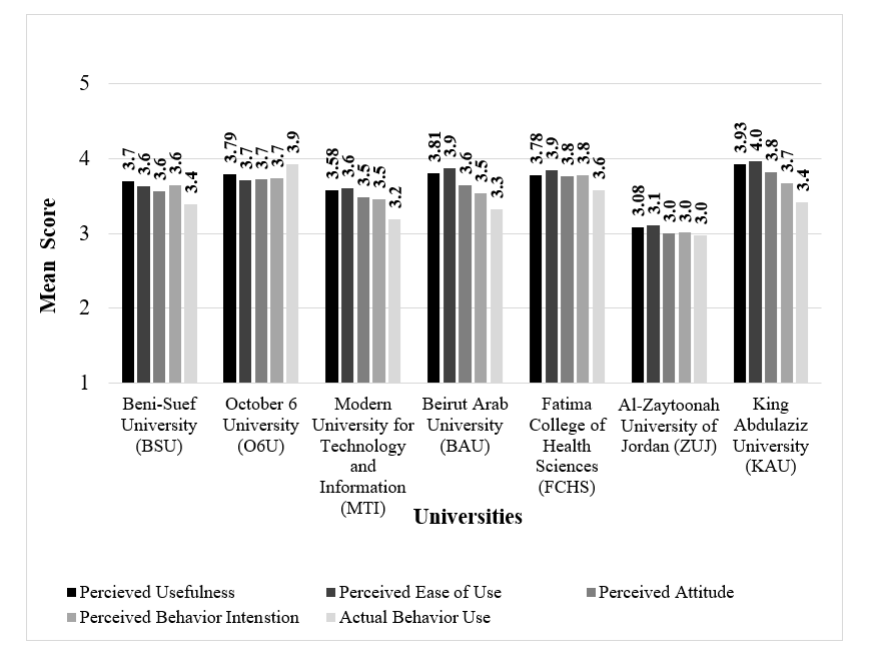
Physiotherapy students’ perception of usefulness, ease of use, attitude, behavior, and actual behavior use of AI-powered chatbots mean scores across universities (N=1066 for all and n=586 for actual behavior use; *P*<.001). AI: artificial intelligence.

In contrast, academic factors had a notable impact. University affiliation significantly influenced the TAM mean score (*P*<.001; Table 4), TAM acceptance categories (*P*<.001; Figure 1), and the 5 constructs mean scores (*P*<.001; Figure 2). CGPA% (Table 3) was positively correlated with the total TAM score (*P*<.001), while academic level was not significantly associated with TAM acceptance categories (*P*=.26).

**Table 4.** Mean total score of physiotherapy students’ acceptance of artificial intelligence–powered chatbots across universities (N=1066).

University	Values, n	Mean score (SD)	Range	*P* value
Fatima College of Health Sciences	107	3.78 (0.70)	1.0‐5.0	<.001
King Abdulaziz University	67	3.77 (0.97)	1.0‐5.0	<.001
October 6 University	200	3.73 (0.79)	1.6‐5.0	<.001
Beirut Arab University	200	3.66 (0.62)	1.4‐5.0	<.001
Beni-Suef University	200	3.60 (0.76)	1.1‐5.0	<.001
Modern University for Technology and Information	200	3.50 (0.85)	1.0‐5.0	<.001
Al-Zaytoonah University of Jordan	92	3.03 (0.91)	1.0‐5.0	<.001
Total	1066	3.59 (0.81)	1.0‐5.0	<.001

Technological factors also showed strong effects. A significant positive correlation was found between prior experience with AI-powered tools beyond chatbots and TAM scores (*P*<.001; Table 2). Additionally, current users of AI-PCs had significantly higher TAM scores (mean 3.89, SD 0.81) than nonusers (mean 3.48, SD 0.79; *P*<.001).

In summary, university affiliation, CGPA%, prior technological experience, and current use were the key factors influencing PT students’ acceptance of AI-PCs. These variables were compared across institutions to further explore their impact.

### University Affiliation and PT Students’ Acceptance of AI-PCs

Significant differences in TAM total scores were observed across universities (*P*<.001; Table 4). ZUJ had the lowest score (mean 3.03), and FCHS the highest (mean 3.78). Post hoc Tukey analysis revealed that ZUJ’s score was significantly lower than all other universities (*P*<.001), while FCHS did not significantly differ from other universities.

TAM acceptance category also varied significantly by universities (*P*<.001; Figure 1). KAU had the highest proportion of students in the high-acceptance category (n=33, 49.3%), whereas ZUJ had the lowest (n=13, 14.1%) and the highest proportion of students in the low-acceptance category (n=9, 9.8%).

Universities also differed significantly in TAM constructs’ scores (*P*<.001; Figure 2 and Table 5). ZUJ had significantly lower PU, PEU, attitude, and BI scores than all other universities (*P*<.001; *P*=.001 for BI vs MTI). It also had the lowest, though nonsignificant, ABU score (2.98). KAU had the highest nonsignificant PU (3.93), PEU (3.96), and attitude (3.82) scores. FCHS had the highest nonsignificant BI score (3.78), while O6U had the highest ABU score (3.93), significantly exceeding all other universities except FCHS (*P*=.42).

**Table 5.** Summary of technology acceptance model (TAM) constructs mean scores of artificial intelligence (AI)–powered chatbot across universities (N=1066).

TAM constructs of AI-powered chatbots	Overall mean score out of 5	*P* value	Highest score university	Lowest score university	Significant Tukey post hoc test	*P* value
Perceived usefulness	3.69	<.001	KAU^a^	ZUJ^b^	ZUJ is lower than all other universities	<.001
Perceived ease of use	3.68	<.001	KAU	ZUJ	ZUJ is lower than all other universities	<.001
Perceived ease of use	3.68	<.001	KAU	ZUJ	MTI^c^ is lower than BAU^d^	.03
Attitude	3.58	<.001	KAU	ZUJ	ZUJ is lower than all other universities	<.001
Behavior intention	3.57	<.001	FCHS^e^	ZUJ	ZUJ is lower than all other universities	<.001 and .001
Behavior intention	3.57	<.001	FCHS	ZUJ	MTI is lower than O6U^f^	.03
Actual behavioral use	3.40	<.001	O6U	ZUJ	O6U is higher than all other universities except FCHS	<.001

a KAU: King Abdulaziz University.

b ZUJ: Al‐Zaytoonah University of Jordan.

c MTI: Modern University for Technology and Information.

d BAU: Beirut Arab University.

e FCHS: Fatima College of Health Sciences.

f O6U: October 6 University.

### CGPA% Across Universities

CGPA% differed significantly among students in the academic levels 2-5 (*P*<.001; Table 6), as level 1 students lacked CGPA data. Post hoc test showed that ZUJ had the lowest CGPA% across all universities (*P*<.001), while BSU recorded the highest. BSU’s CGPA% was significantly higher than ZUJ, BAU, FCHS (*P*<.001 for each), and MTI (*P*=.002) and higher, though nonsignificant, than KAU (*P*=.10) and O6U (*P*=.10). Additionally, BAU had significantly lower CGPA% than KAU (*P*<.001), MTI (*P*=.03), and O6U (*P*=.001). FCHS also had significantly lower CGPA% than BSU, KAU (*P*<.001 each), MTI (*P*=.04), and O6U (*P*=.003).

**Table 6.** Comparison of physiotherapy students’ mean cumulative grade point average percentage (CGPA%) and previous technological experience across universities.

Universities	CGPA%			Previous technological experience (1‐5 scale)		
	Students, n	Mean (SD)	*P* value	Students, n	Mean (SD)	*P* value
Al-Zaytoonah University of Jordan	92	67.2 (17.5)	<.001	92	2.4 (1.6)	<.001
Beirut Arab University	163	76.3 (14.0)	<.001	200	3.3 (1.4)	<.001
Beni-Suef University	171	86.0 (10.6)	<.001	200	3.1 (1.4)	<.001
Fatima College of Health Sciences	80	75.4 (10.4)	<.001	107	3.5 (1.3)	<.001
King Abdulaziz University	65	84.9 (7.5)	<.001	67	3.5 (1.5)	<.001
Modern University for Technology and Information	186	80.6 (12.8)	<.001	200	3.1 (1.5)	<.001
October 6 University	167	82.2 (14.0)	<.001	200	2.8 (1.6)	<.001
Total	924	79.7 (14.0)	<.001	1066	3.1 (1.5)	<.001

### Previous Technological Experience Across Universities

PT students’ prior experience with AI tools beyond chatbots varied significantly across universities (*P*<.001; Table 6). ZUJ students had the lowest prior experience, significantly lower than all other universities (*P*<.001). Additionally, MTI students had significantly less prior experience than FCHS students (*P*=.04).

### Current Use of AI-PCs Across Universities

PT students’ use of AI-PCs differed significantly across universities (*P*<.001). KAU students reported the highest use rate (n=56, 83.6%), while FCHS had the lowest (n=34, 31.8%; Table 7).

**Table 7.** Physiotherapy students’ use of artificial intelligence (AI) chatbots across universities (N=1066).

Use of AI chatbots	Beni-Suef University, n (%)	October 6 University, n (%)	Modern University for Technology and Information, n (%)	Fatima College of Health Sciences, n (%)	King Abdulaziz University, n (%)	Beirut Arab University, n (%)	Al‐Zaytoonah University of Jordan, n (%)	Total, n (%)
Users	117 (58.5)	89 (44.5)	129 (64.5)	34 (31.8)	56 (83.6)	120 (60)	41 (44.6)	586 (55)
Nonusers	83 (41.5)	111 (55.5)	71 (35.5)	73 (68.2)	11 (16.4)	80 (40)	51 (55.4)	480 (45)
Total	200 (100)	200 (100)	200 (100)	107 (100)	67 (100)	200 (100)	92 (100)	1066 (100)

### Key Predictors of AI-PCs’ Acceptance Among PT Students

A multiple linear regression was conducted to identify predictors of AI-PCs’ acceptance among PT students. To meet regression analysis requirements, university affiliation was converted to dummy variables (ZUJ as the reference group), and current use was coded numerically. The model was statistically significant (*F*_9,914_=32.33; *P*<.001), with an *R*² of 0.24, indicating that 24% of the variance in TAM score was explained. Significant predictors included prior experience with AI-PCs (B=0.23; *P*<.001) and university affiliation. Students from all universities scored significantly higher than those from ZUI, with the strongest effects seen at O6U (B=0.59; *P*<.001), FCHS (B=0.44; *P*<.001), and BAU (B=0.42; *P*<.001). CGPA% and current use were not significant predictors (*P*=.23 and *P*=.10, respectively; Table 8).

**Table 8.** Regression analysis results: predictor factors of physiotherapy students’ acceptance of artificial intelligence–powered chatbots (N=1066).^a^

Predictor	B (unstandardized coefficient)	SE	β (standardized coefficient)	*t* test (*df*)	*P* value
Constant	2.33	0.15	0.00	15.90 (2)	<.001
Beni-Suef University (BSU_dummy)	0.36	0.10	0.17	3.63 (2)	<.001
October 6 University (O6U_dummy)	0.59	0.10	0.27	6.00 (2)	<.001
Modern University for Technology and Information (MTI_dummy)	0.27	0.10	0.13	2.82 (2)	<.001
Beirut Arab University (BAU_dummy)	0.40	0.10	0.19	4.16 (2)	<.001
Fatima College of Health Sciences (FCHS_dummy)	0.44	0.11	0.15	3.86 (2)	<.001
King Abdulaziz University (KAU_dummy)	0.45	0.12	0.14	3.65 (2)	<.001
Prior experience	0.23	0.02	0.43	13.93 (2)	<.001
CGPA%^b^	0.0	0.0	0.04	1.21 (2)	.23
Use	0.00	0.05	0.00	0.01 (2)	.99

a Al Zaytoonah University of Jordan is the reference group.

b CGPA%: cumulative grade point average percentage.

### Regression Equations

#### Full Model (All Predictors)

TAM score=2.33+0.00 (use)+0.23 (prior experience)+0.36 (BSU)+0.27 (MTI)+0.42 (BAU)+0.44 (FCHS)+0.45 (KAU)+0.59 (O6U)+0.00 (CGPA%)

#### Simplified Model (Significant Predictors Only)

TAM score=2.33+0.23 (prior experience)+0.36 (BSU)+0.27 (MTI)+0.42 (BAU)+0.44 (FCHS)+0.45 (KAU)+0.59 (O6U)

(Note: To calculate a student’s score, assign “1” to their university and “0” to all others.)

Previous experience emerged as the strongest predictor (standardized β=.43), followed by O6U (β=0.27), indicating that students’ prior experience with AI tools had a larger impact on their acceptance score than university affiliation.

## Discussion

### Principal Findings

PT students demonstrated a moderate acceptance of AI-PCs. University affiliation, CGPA%, prior technological experience, and current use were identified as external factors influencing students’ acceptance. However, the regression model confirmed that students’ prior technological experience is the strongest predictor.

AI-PCs’ use is steadily increasing in university education, with students’ acceptance playing a key role in their adoption. For PT students, the clinical and practical demands of their curriculum present unique challenges to AI-PCs’ acceptance. This study addressed the need to assess undergraduate PT students’ acceptance of AI-PCs in their studies and identify personal, academic, and technological factors that influence it.

The multisite design enhances validity and generalizability, offering a broad view of AI-PCs’ acceptance among PT students. Including all academic levels captures diverse perceptions, while a mean CGPA% of 79.7% reflects an average profile, minimizing academic performance–related bias.

Despite the rigorous nature of PT programs—with their intensive practical and clinical components—PT students still showed a moderate level of acceptance toward AI-PCs. This suggests a continued openness to AI-PCs, even within a rigorous academic environment. A study was conducted in South Korea among medical students and physicians, and it revealed that although physicians were cautious about the use of AI-PCs, particularly ChatGPT, in guiding patients’ treatment, students had a positive perception of using ChatGPT for guiding treatment and medical education [31], which supports the concept that students might be more open to using advanced technology.

TAM constructs’ scores provided deeper insight into the PT students’ acceptance of AI-PCs. All scores fell within the moderate range, with PU and PEU scoring the highest means, indicating that PT students perceived AI-PCs as both useful and easy to use. However, slightly lower scores for attitude and BI suggest that while students acknowledge these benefits, their enthusiasm and intent to adopt AI-PCs are still developing. The lowest score for ABU highlights limited real-world use, reinforcing the study’s overall finding of moderate acceptance.

In line with TAM’s 3-stage process [21], external factors play a crucial role in shaping students’ acceptance of AI-PCs. The first stage includes PU and PEU, which reflect students’ perceptions of usefulness and ease of use [26,27]. These influence attitude, which may substitute for BI [21,28], forming the second stage. Together, PU, PEU, attitude, and BI predict ABU [23]. Personal, academic, and technological factors influence students’ perceptions, attitudes, and adoption behaviors toward AI technologies [6] such as AI-PCs. Among all the external factors, predictors, prior experience with AI-powered tools and applications beyond chatbots, and university affiliation had the strongest predictive influence on students’ acceptance of AI-PCs.

Older students might be expected to show greater acceptance of AI-PCs due to increased exposure through academic progression and peer networks. Conversely, younger students, particularly Generation Z, could also be more accepting, having grown up immersed in digital technology. Similarly, male students are often presumed to show higher acceptance due to greater interest in technology and gaming. However, the findings did not support these assumptions, as age, sex, and academic level showed no significant correlation with AI-PCs’ acceptance. Several hypotheses may explain this. First, institutional integration of AI tools may provide equal exposure to all students, minimizing demographic differences. Second, although Gen Z is highly technology competent, their adoption of AI-PCs may depend on perceived academic relevance. Third, sex-based assumptions about technology enthusiasm may not translate into actual academic tool use.

Students with greater exposure to AI tools demonstrated higher acceptance of AI-PCs, a logical outcome, as familiarity builds confidence. For these students, chatbots were a natural extension of technologies they already use, making adoption more intuitive. Supporting this, Horowitz et al [32] found that individuals with more familiarity and expertise in AI were more likely to support autonomous technologies than those with limited understanding, suggesting that experience enhances acceptance of new technologies.

University affiliation emerged as a key predictor of AI-PCs’ acceptance. ZUJ recorded the significantly lowest total TAM score and the significantly lowest scores in 4 of the 5 constructs (except ABU). It also had the smallest proportion of students in the high-acceptance category and the highest proportion in the low-acceptance group, raising the question: What explains ZUJ’s lower acceptance?

One possible factor is CGPA%, as ZUJ had the lowest CGPA%. However, CGPA% alone is unlikely to explain the outcome. For example, BSU, despite having the highest CGPA%, ranked the third lowest in TAM and the fourth lowest in the proportion of high-acceptance students. This suggests that academic performance is not a decisive factor in acceptance.

Prior technological experience, however, appears more influential. ZUJ students had the lowest prior technological experience across all universities, which aligns with their low acceptance rate. While prior experience was notably higher in the 3 universities with better acceptance (FCHS, KAU, and O6U), it was also relatively low in universities with low acceptance, such as BSU and MTI.

Although academic level was not significantly correlated with acceptance, most participating ZUJ students were in levels 1 to 3, as nearly all level 4 students were nonnationals and thus excluded. Additionally, ZUJ’s PT department was established only 4 years ago. Together, these factors likely contributed to students’ limited prior technological experience, and consequently, their lower acceptance of AI-PCs.

In contrast, examining the current use, ZUJ showed a moderate percentage of users, like O6U, the only university with a significantly higher score in one of the constructs. Meanwhile, FCHS demonstrated the lowest percentage of students currently using AI-PCs. If current use was a key factor in acceptance, both FCHS and O6U would be expected to have a high use rate, which was not the case. These patterns suggest that current use is not a reliable predictor of acceptance.

Hence, linear regression analysis was essential to clarify which external factors significantly predict PT students’ acceptance of AI-PCs. Although the model explained 24% of the variance, it identified prior technological experience as the strongest predictor, confirming earlier assumptions about its importance. The analysis also confirmed that CGPA% and current use were not significant predictors and were excluded from the regression equation. In addition to prior experience, university affiliation emerged as an external predictor, though with a smaller effect size. These results confidently attribute ZUJ’s low acceptance to its students’ limited prior technological experience.

While the findings highlight university affiliation as a key predictor of acceptance, this study did not elaborate on the detailed cultural characteristics of studentship within each country or institution. Cultural norms, instructor-student dynamics, technological infrastructure, and pedagogical styles can certainly influence students’ attitudes toward innovation. However, including a comprehensive cultural profile for each country or institution would have lengthened the paper unnecessarily. To reduce cultural bias and isolate academic and technological factors, only national students were recruited. This approach served to eliminate variability related to cross-national cultural differences, providing a more homogeneous sample to assess institutional influence on AI chatbot acceptance.

Interestingly, construct-level results revealed 2 cases where students’ ABU did not align with their PU or PEU. These discrepancies suggest gaps between perceptions and actual use of AI-PCs or the influence of external factors on behavior. Supporting this, a study of 399 students in Hong Kong revealed that while attitudes toward AI technologies were generally positive, actual use remained limited, highlighting a gap between favorable perception and limited actual use of AI chatbots in education [33]. Another example is a study conducted in the United Arab Emirates among 265 recently graduated medical students, which reported an overall positive attitude and optimism toward the future of AI in medical education and health care but also revealed limited use of AI-PCs [34]. Similarly, a national survey of 693 medical students across 57 Chinese universities reported that only 28.7% used AI chatbots for studying, despite 91% acknowledging their usefulness for accessing medical information, reinforcing the disconnect between intention and practice [35]. Specifically, O6U demonstrated the highest ABU across all universities, despite reporting only moderate PU and PEU scores. In other words, O6U students used AI-PCs in their studying, even though they perceived them as only moderately useful or easy to use. Several external factors may explain this pattern. One possibility is a university mandate, such as a course assignment, which may have driven use regardless of personal perception. Another is social influence, where encouragement from peers or faculty members promotes greater use. A third explanation could be habituation exposure; students may have become accustomed to using the AI-PCs through repeated encounters, resulting in higher ABU despite lower PU and PEU.

In contrast, KAU students showed the opposite pattern, high PU and PEU scores but lower ABU. This highlights the common gap between what students perceive and what they do, especially when technology is available but not fully integrated into the learning process. In this case, positive perceptions of AI-PCs were not sufficient to drive consistent use in academic practices. A large-scale study, which analyzed responses from over 34,000 college students, supports this pattern, showing that favorable views of educational technology did not always result in increased use, particularly when the tools were not embedded in instruction [36].

Findings from O6U and KAU highlight the complexity of technology acceptance and the influence of external factors beyond individual perceptions. They illustrate how such external factors can sometimes override personal perceptions in driving actual behavior, warranting further exploration.

### Limitation

While this study examined selected personal, academic, and technological factors, many other factors may contribute to PT students’ acceptance of AI-PCs. These include students’ learning style, personality traits, academic load, technology anxiety, self-regulated learning skills, language proficiency, faculty support, and access to physical resources such as internet connectivity.

### Further Work

While this study focused on personal, academic, and technological factors influencing PT students’ AI-PCs, several broader contextual variables warrant exploration in future research. These include deeper investigations into the culture of studentship, both within and across countries in the region, such as how students interact with instructors and technology, their expectations, and the learning environments they are embedded in. Additionally, variations in the structure of PT programs, such as 4- versus 5-year tracks, patterns of matriculation, and progression rates, could provide further insight into students’ readiness and attitudes toward adopting educational technologies. Future studies may also benefit from considering national regulations and licensing frameworks, which shape the continuum of PT education, including the integration of internship, postgraduate training, and continuing education. These elements, although beyond our study’s scope, are crucial for developing a comprehensive understanding of technology acceptance within the broader context of PT education.

### Conclusions

PT students demonstrated a moderate acceptance of AI-PCs. University affiliation, CGPA%, prior technological experience, and current use were identified as external factors influencing students’ acceptance. However, the regression model confirmed that prior technological experience is the strongest predictor of AI-PCs’ acceptance among PT students.

## Abbreviations

ABU: actual behavioral use
AI: artificial intelligence
AI-PC: artificial intelligence–powered chatbot
BAU: Beirut Arab University
BI: behavioral intention
BSU: Beni-Suef University
CGPA: cumulative grade point average
CGPA%: cumulative grade point average percentage
CHERRIES: Checklist for Reporting Results of Internet E-Surveys
FCHS: Fatima College of Health Sciences
KAU: King Abdulaziz University
MTI: Modern University for Technology and Information
O6U: October 6 University
PEU: perceived ease of use
PT: physiotherapy
PU: perceived usefulness
STROBE: Strengthening the Reporting of Observational Studies in Epidemiology
TAM: technology acceptance model
ZUJ: Al‐Zaytoonah University of Jordan
